# Effect of self-reported walking difficulty on bone mass and bone resorption marker in Japanese people aged 40 years and over

**DOI:** 10.1186/s40101-016-0114-6

**Published:** 2016-10-11

**Authors:** Yasuyo Abe, Takayuki Nishimura, Kazuhiko Arima, Mitsuo Kanagae, Satoshi Mizukami, Yoshihito Tomita, Takuhiro Okabe, Hisashi Goto, Itsuko Horiguchi, Kiyoshi Aoyagi

**Affiliations:** 1 Department of Public Health, Nagasaki University Graduate School of Biomedical Sciences, Nagasaki, Japan; 2 Department of Rehabilitation, Nishi-Isahaya Hospital, Isahaya, Japan; 3 Goto Health Care Office, Nagasaki, Japan; 4 Center for Public Relations Strategy, Nagasaki University, Nagasaki, Japan

**Keywords:** Walking difficulty, Bone mass, Calcaneal stiffness index, Bone turnover

## Abstract

**Background:**

This study aimed to examine the association of walking difficulty with bone mass or bone turnover among community-dwelling Japanese people aged 40 years and older.

**Methods:**

We studied 1097 community-dwelling Japanese people aged 40 years and older (379 men and 718 women) who were invited to participate in periodic health examinations in 2006–2009. Walking difficulty was defined as having difficulty walking 100 m on a level surface (self-administered questionnaire). Calcaneal stiffness index (bone mass) was measured by quantitative ultrasound. Spot urine samples were collected, and urinary N-terminal cross-linking telopeptide of type I collagen (NTx) was measured. Values were corrected for creatinine (Cre) concentration.

**Results:**

The prevalence of walking difficulty was significantly higher in women than in men (7.4 vs. 3.4 %, *p* = 0.011) and significantly increased with age in men (*p* for trend = 0.02) and women (*p* for trend <0.001). In univariate analysis, men and women with walking difficulty were older (*p* < 0.001) and had a lower stiffness index (*p* < 0.001), compared with those without walking difficulty. Among women, individuals with walking difficulty had significantly higher urinary NTx/Cre than those without walking difficulty (*p* < 0.001); however, this was not so among men (*p* = 0.39). Multiple regression analysis adjusted for age, weight, and menopausal status showed a significant association between walking difficulty and lower stiffness index in men (*p* = 0.004) and women (*p* = 0.005). In women, walking difficulty was significantly associated with higher NTx/Cre (*p* = 0.001), but not in men (*p* = 0.35).

**Conclusions:**

Walking difficulty may contribute to low bone mass in both sexes but might cause high bone turnover in women only.

## Background

Low bone mass and accelerated bone turnover contribute to osteoporotic fractures. Quantitative ultrasound (QUS) parameters of the calcaneus predict fracture risk among elderly men and women [1–5]. Biochemical markers of bone turnover predict fracture risk in elderly women [6–9]. Cross-linked N-telopeptide of type I collagen (NTx) is one of the biochemical markers of bone resorption and is widely used in epidemiological [6, 7] and clinical studies [10].

Physical activity influences bone mass and bone turnover. High levels of physical activity are associated with high QUS parameters among elderly women [11–16]. Increased physical activity is effective in reducing the levels of bone resorption markers among elderly men and women [17–20].

Walking ability is an important element of physical activity. Thus far, the effect of walking difficulty on bone mass or bone turnover has not well been studied. Kitagawa et al. [21] reported the association between levels of walking activity and levels of QUS parameters or bone turnover in elderly women, but there are no data in men. Chen et al. [22] reported the association between measures of mobility (graded using a four-point scale as follows: walks unaided, uses stick, uses frame, needs wheelchair) and bone turnover markers among older people in residential care facilities, but results were not shown separately for men and women.

The self-reported walking difficulty assessed by asking people if they have any difficulty walking 100 m on a level surface [23] is considered to detect moderate immobility [24]. This simple and easy to apply question has been applied in many aging studies [23, 25, 26]. It would be necessary to examine the association of walking difficulty with bone health status in order to evaluate their physiological adaptations to moderate immobility.

The purpose of this study was to assess the association of walking difficulty (chronic immobility) with bone mass and bone turnover among community-dwelling Japanese men and women aged 40 years and older.

## Methods

Subjects were community-dwelling people aged 40 years and older at Nagasaki Prefecture, Japan, who were invited to participate in periodic health examinations in 2006–2009. A total of 1097 people (379 men and 718 women) participated in the study. Subjects were asked if they had difficulty walking 100 m on a level surface (walking difficulty). Calcaneal stiffness index (bone mass) was measured using a Lunar Achilles device (GE Lunar Corp., Madison, WI). Spot urine samples (8:00–10:00 am) were collected. Urinary N-terminal cross-linking telopeptide of type I collagen (NTx), a marker of bone resorption, was measured using an enzyme immunoassay. Values were corrected for creatinine (Cre) concentration. Height (m) and weight (kg) were measured with light clothing and without shoes, and body mass index (BMI) was calculated as weight (kg)/height^2^ (m^2^).

Written informed consent was obtained from all the subjects prior to the study. The ethics committee of Nagasaki University Graduate School of Biomedical Sciences approved the study.

### Statistical analysis

The student *t* test was used to determine the significance of differences between subjects with and without walking difficulty. Cochran-Armitage trend test was used to evaluate differences in the prevalence of walking difficulty among age groups (40–49, 50–59, 60–69, 70–79, and 80 and over). Because urinary NTx/Cre had a skewed distribution, logarithmic transformation was performed for the analysis. Student’s *t* test was used to examine the difference in variables between individuals with and without walking difficulty. Multiple linear regression analysis was used to explore the effects of walking difficulty on calcaneal stiffness index or urinary NTx/Cre, adjusting for age, weight, and menopausal status. Data were analyzed using the Statistical Analysis System Version 9.2. (SAS Institute Inc. Cary, NC).

## Results

Table 1 shows characteristics of subjects. Mean age (SD) was 65.6 (9.8) years in men and 65.0 (9.6) years in women. The prevalence of walking difficulty was significantly higher in women than in men (7.4 vs. 3.4 %, *p* = 0.011).

**Table 1:** Characteristics of study subjects

	Men (*n* = 379)	Women (*n* = 718)
	Mean (SD)	Mean (SD)
Age (years)	65.6 (9.8)	65.0 (9.6)
Weight (kg)	62.7 (9.1)	52.6 (8.4)
Height (cm)	162.9 (6.1)	150.7 (6.0)
Body mass index (kg/m^2^)	23.6 (2.9)	23.1 (3.4)
Calcaneal stiffness index	86.5 (17.4)	69.9 (12.3)
Urinary NTx/Cre (nmol BCE/mmol•CRE)	36.2 (16.9)	56.6 (26.8)
	Number (%)	Number (%)
Walking difficulty	13 (3.5)	53 (7.4)*
Postmenopausal	–	655 (91.2)

*NTx* N-terminal cross-linking telopeptide of type I collagen, *Cre* creatinine, *BCE* bone collagen equivalents

*p = 0.011 vs. men

Table 2 shows prevalence of walking difficulty by age groups. The prevalence of walking difficulty significantly increased with age both in men (*p* for trend = 0.02) and in women (*p* for trend <0.001).

**Table 2:** Prevalence of walking difficulty by age group

Age (years)	40–49	50–59	60–69	70–79	80 and over	*p* for trend^a^
	Men					
	(*n* = 24)	(*n* = 70)	(*n* = 139)	(*n* = 119)	(*n* = 27)	
	0 (0.0)	2 (2.9)	2 (1.4)	6 (5.0)	3 (11.1)	0.02
	Women					
	(*n* = 54)	(*n* = 146)	(*n* = 274)	(*n* = 213)	(*n* = 31)	
	0 (0.0)	3 (2.1)	18 (6.6)	24 (11.3)	8 (25.8)	<0.0001

Values are number (%)

^a^Cochran-Armitage trend test

In univariate analysis, men and women with walking difficulty were significantly older (*p* = 0.0008 in men, *p* < 0.0001 in women) and had a significantly lower calcaneal stiffness index (*p* = 0.0006 in men, *p* < 0.0001 in women) compared with those without walking difficulty (Table 3). Among women, individuals with walking difficulty had significantly higher urinary NTx/Cre than those without walking difficulty (*p* < 0.0001), but no significant association was observed among men (*p* = 0.39).

**Table 3:** Comparison of variables between subjects with and without walking difficulty

	With	Without	*p* ^a^
	Men		
	(*n* = 13)	(*n* = 366)	
Age (years)	72.6 (9.0)	65.4 (9.7)	0.0008
Weight (kg)	62.0 (9.6)	62.7 (9.1)	0.78
Body mass index (kg/m^2^)	24.2 (3.6)	23.6 (2.9)	0.41
Calcaneal stiffness index	70.2 (13.4)	87.1 (17.3)	0.0006
Log (NTx/Cre) (nmol BCE/mmol•CRE)	3.6 (0.53)	3.5 (0.43)	0.39
	Women		
	(*n* = 53)	(*n* = 665)	
Age (years)	72.1 (8.1)	64.4 (9.5)	<0.0001
Weight	51.9 (8.9)	52.7 (8.4)	0.53
Body mass index (kg/m^2^)	24.0 (3.5)	23.1 (3.4)	0.07
Calcaneal stiffness index	60.0 (13.1)	70.7 (14.1)	<0.0001
Log (NTx/Cre) (nmol BCE/mmol•CRE)	4.2 (0.48)	3.9 (0.48)	<0.0001

*NTx* N-terminal cross-linking telopeptide of type I collagen, *Cre* creatinine, *BCE* bone collagen equivalents

^a^ *t* test

Multiple linear regression analysis adjusted for age, weight, and menopausal status showed a significant association between walking difficulty and lower calcaneal stiffness index in men (*p* = 0.004) and women (*p* = 0.005) (Table 4). In women, walking difficulty was significantly associated with higher NTx/Cre (*p* = 0.001), but not in men (*p* = 0.35).

**Table 4:** Association of walking difficulty to calcaneal stiffness index or urinary NTx/Cre: results of multiple regression analysis adjusting for age, weight, and menopausal status

Outcome	Estimate	Standard error	*p*
Men			
Calcaneal stiffness index	−13.7	4.7	0.004
Log (NTx/Cre)	0.11	0.12	0.35
Women			
Calcaneal stiffness index	−4.7	1.7	0.005
Log (NTx/Cre)	0.22	0.07	0.001

*NTx* N-terminal cross-linking telopeptide of type I collagen, *Cre* creatinine

## Discussion

We showed that walking difficulty (having difficulty walking 100 m on a level surface) was associated with a lower calcaneal stiffness index (bone mass) in both sexes and with higher NTx/Cre (bone resorption) only in women. Bone metabolism status might differ between men and women. To the best of our knowledge, this is the first study to examine the association of walking difficulty (chronic immobility) with bone resorption markers separately for men and women.

Physical activity contributes to high QUS parameters [1–5]. Kitagawa et al. [21] showed that number of daily walking steps was positively associated with QUS parameters (speed of sound, broadband ultrasound attenuation, or stiffness index) among postmenopausal Japanese women. Sun et al. [27] showed that gait parameters were associated with calcaneal stiffness index among community-dwelling Japanese elderly women. We showed a significant association between walking difficulty and lower calcaneal stiffness index in both sexes, suggesting that chronic moderate immobility may influence low bone mass in men and women.

Chronic immobility could markedly affect bone metabolism in elderly people [28]. Sorva et al. [28] found that serum carboxyterminal cross-linked telepeptide of type I collagen, a bone resorption marker, was elevated in bedridden elderly subjects. Bischoff et al. [29] found that among residents of a long-stay geriatric ward, more immobile subjects had significantly higher excretion rates for urinary deoxypyridinoline, a bone resorption marker. On the other hand, Chen et al. [22] found that in elderly men and women living in residential aged care facilities, age- and gender-adjusted levels of serum carboxyterminal telopeptide of type I collagen, a bone resorption marker, were significantly higher in men and women with poorer mobility, but no significant association was found after further adjustment for weight, parathyroid hormone, and other covariates. Thus, the relationship between chronic immobility and bone resorption markers is controversial. Furthermore, few studies have assessed men separately, and the effect of relatively moderate immobility on bone resorption markers has not yet been determined. Further study is needed to elucidate the effect of moderate immobility on bone resorption markers, considering possible differences between men and women.

This study has several limitations. First, because this study was conducted in a cross-sectional setting, these results do not show a causal relationship. Longitudinal studies are required to confirm whether changes in QUS parameters and NTx levels are affected by walking difficulty. Second, study participants were mobile enough to attend the examination site, contributing to selection bias. Third, potential confounders that can affect immobility and increased bone resorption, such as lifestyle factors (smoking, alcohol consumption, and exercise), vitamin D deficiency, low calcium intake, secondary hyperparathyroidism, current medications, years after menopause, and renal insufficiency [22], were not available in this study.

In conclusion, walking difficulty may contribute to low bone mass in both sexes but might cause high bone turnover only in women. Our findings suggest that chronic immobility is an important factor contributing to the low bone mass and high bone turnover especially in women. Studies of bone mass and biochemical markers of bone turnover in various at-risk populations should also consider the influence of mobility.

## Abbreviations

BCE: Bone collagen equivalents
BMI: Body mass index
Cre: Creatinine
NTx: Urinary cross-linked N-telopeptide of type I collagen
QUS: Quantitative ultrasound
SD: Standard deviation
